# Proteomics and Lipidomics in Inflammatory Bowel Disease Research: From Mechanistic Insights to Biomarker Identification

**DOI:** 10.3390/ijms19092775

**Published:** 2018-09-15

**Authors:** Bjoern Titz, Raffaella M. Gadaleta, Giuseppe Lo Sasso, Ashraf Elamin, Kim Ekroos, Nikolai V. Ivanov, Manuel C. Peitsch, Julia Hoeng

**Affiliations:** 1 PMI R&D, Philip Morris Products S.A., Quai Jeanrenaud 5, CH-2000 Neuchatel, Switzerland; RaffaellaMaria.Gadaleta@pmi.com (R.M.G.); Giuseppe.LoSasso@pmi.com (G.L.S.); Ashraf.Elamin@pmi.com (A.E.); Nikolai.Ivanov@pmi.com (N.V.I.); Manuel.Peitsch@pmi.com (M.C.P.); 2 Lipidomics Consulting Ltd., Irisviksvägen 31D, 02230 Esbo, Finland; kim@lipidomicsconsulting.com

**Keywords:** inflammatory bowel disease, proteomics, lipidomics, biomarkers, personalized medicine

## Abstract

Inflammatory bowel disease (IBD) represents a group of progressive disorders characterized by recurrent chronic inflammation of the gut. Ulcerative colitis and Crohn′s disease are the major manifestations of IBD. While our understanding of IBD has progressed in recent years, its etiology is far from being fully understood, resulting in suboptimal treatment options. Complementing other biological endpoints, bioanalytical “omics” methods that quantify many biomolecules simultaneously have great potential in the dissection of the complex pathogenesis of IBD. In this review, we focus on the rapidly evolving proteomics and lipidomics technologies and their broad applicability to IBD studies; these range from investigations of immune-regulatory mechanisms and biomarker discovery to studies dissecting host–microbiome interactions and the role of intestinal epithelial cells. Future studies can leverage recent advances, including improved analytical methodologies, additional relevant sample types, and integrative multi-omics analyses. Proteomics and lipidomics could effectively accelerate the development of novel targeted treatments and the discovery of complementary biomarkers, enabling continuous monitoring of the treatment response of individual patients; this may allow further refinement of treatment and, ultimately, facilitate a personalized medicine approach to IBD.

## 1. Introduction

**Figure 1:**
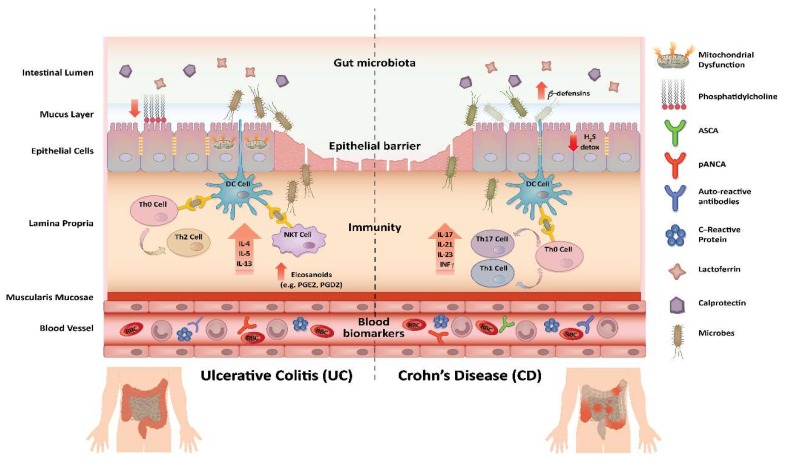
Pathomechanisms and selected biomarkers of IBD. The main pathogenesis-associated changes are shown for UC and CD. For both diseases, changes in gut microbiota, disruption of the epithelial barrier function, and chronic immune-activation are observed. Differences have also been reported, such as for immune-regulatory processes and epithelial effects. Biomarkers can be measured directly in tissue biopsies or upon release into the gut (e.g., fecal calprotectin) or the blood (e.g., autoreactive antibodies).

Inflammatory bowel disease (IBD) represents a group of chronic intestinal disorders that is characterized by recurrent inflammation affecting the gastrointestinal tract. Ulcerative colitis (UC) and Crohn′s disease (CD) are the two main clinically defined manifestations of IBD, each with distinctive clinical and pathological features (Figure 1) [1].

UC is a chronic, non-transmural, inflammatory disease that is characterized by diffuse mucosal inflammation involving the colon. UC lesions generally begin in the rectum and extend proximally in an uninterrupted pattern, involving part or all of the colon [2]. The main clinical symptom of UC is bloody diarrhea; extra-intestinal manifestations are common, including musculoskeletal, dermatological, ocular, and hepatobiliary co-morbidities. The clinical progression of UC is characterized by exacerbations and remissions [3,4,5]. From an immunological standpoint, UC present with an atypical T helper cell (Th) 2 response involvement, mediated by secretion of interleukin (IL)-13 by natural killer T cells [6,7,8]. 

CD is a relapsing, transmural, inflammatory disease that may affect the entire gastrointestinal tract, from the oral cavity to the rectum. It is characterized by a non-contiguous inflammatory pattern with areas of uninflamed mucosa. Its primary clinical symptom is abdominal pain with weight loss; it can also present with extra-intestinal co-morbidities similar to those described in UC patients. T cell profiles in CD and UC differ, and Th1 cytokine profiles are dominant in CD [3,4,9,10,11,12]. 

With its intrinsic multifactorial etiology, IBD springs from an altered interaction between the resident intestinal flora and the immune mucosal cells, due to the influence of environmental factors affecting genetically susceptible hosts. This generates a vicious cycle in which the intestinal epithelium loses its inherent integrity and becomes more pervious, priming a positive feedback loop that involves increased exposure to the intestinal microbiota, thereby leading to an uncontrolled inflammatory response [13,14,15,16].

The entangled nature of all factors contributing to its pathogenesis and their reciprocal influence led to the conceptual framing of these factors as the IBD interactome, which encompasses aspects of the immunome, microbiome, exposome, and genome [17,18,19,20,21]. In the gut microenvironment, where the multitude of IBD components functionally interact, no information about a single molecule, single gene, or single microbe can sufficiently explain the collective events that result from constant feedback and feed-forward signaling. For this reason, along with the broad variability of biological signatures in humans, an inclusive comprehension of how every component of the IBD interactome acts on and influences other components is imperative.

Unravelling the connection between known and unknown paths is needed to untangle all levels of the IBD interactome, and omics approaches—interpreted within systems biology analysis frameworks—could greatly accelerate this mission. Omics techniques allow the study of related sets of biological molecules in a comprehensive fashion; the development and use of these techniques have been rising sharply in recent decades [22]. Omics techniques include genomics, transcriptomics, metabolomics, proteomics, and lipidomics. While genetic and transcriptomic studies have been explored thoroughly in the context of IBD, proteomics and lipidomics research is now emerging as a new frontier to investigate these diseases. 

In addition to yielding insights into complex etiologies, omics techniques are commonly applied for the discovery of biomarkers to support diagnosis, stratification, and treatment monitoring. For instance, although endoscopy remains the gold standard for the diagnosis and monitoring of IBD, the use of molecular biomarkers in clinical practice, including fecal calprotectin [23], serum C-reactive protein (CRP) [24], and serum autoantibodies, has been extremely important and supportive. However, their low sensitivity and high variability characteristics limit clinical efficacy [25,26]. Thus, IBD clinical management would clearly benefit from the identification of novel molecular biomarkers [27,28], which could provide less invasive assessment methods, facilitate novel targeted treatments, and decrease the medical financial burden [24,29,30]. 

In this review, we focus on the application of omics technologies, particularly proteomics and lipidomics, for IBD research. Driven by recent advances in mass spectrometry (MS) instrumentation, both omics approaches are rapidly evolving and could provide deeper insights into the complex pathogenesis of IBD [31,32]. 

## 2. IBD Diagnosis and Treatment: Need for Novel Approaches

CD or UC diagnosis integrates disease symptoms with data from endoscopic and histological evaluations. Treatment choices are often driven by symptom severity and patient responsiveness in order to induce remission and prevent occurrence of flares. Available IBD treatments include mesalazine, corticosteroids, immunosuppressive drugs, and monoclonal antibodies against tumor necrosis factor alpha (TNF-α) [33,34]. However, approximately one-third of patients do not exhibit improvement after induction therapy (primary non-response); loss of response may occur gradually in up to 20% of patients per year [35,36], and selection of alternative treatment strategies in non-responsive patients is extremely challenging. Importantly, treatment success depends on several patient-specific factors, including the optimization of therapeutic dosage, co-morbidities, and use of the correct disease activity index [37]. In the future, it is expected that—based on a deeper mechanistic understanding of the disease—novel molecularly targeted therapies, embedded in adaptive personalized medicine treatment frameworks, are likely to bring further therapeutic benefits to the field [38,39]. Clearly, such personalized medicine approaches will rely strongly on the availability of effective molecular companion diagnostics.

**Figure 2:**
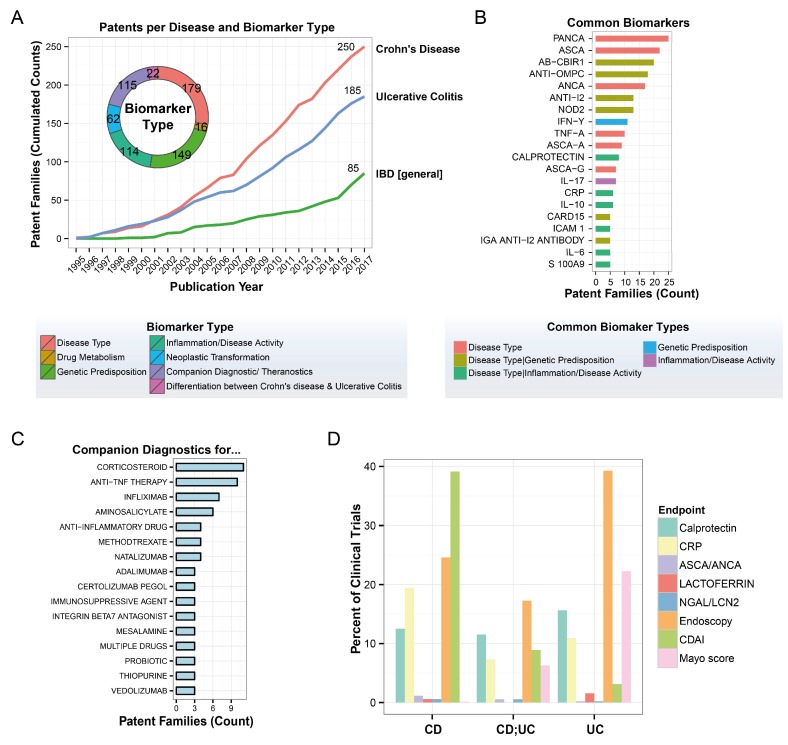
Patent survey for IBD biomarkers. (**A**) Development of IBD biomarker patents over the last two decades. A worldwide IBD patent search for the last 20 years was conducted by using the Questel Orbit database. Cumulative numbers of patent families for CD, UC, and general IBD over time are shown in the line chart. The donut chart shows the current breakdown of number of patents by biomarker type. Note that a patent family can be associated with more than one disease and biomarker type. (**B**) Top 20 IBD biomarker patent families. The color code shows the commonly associated disease pattern for each biomarker. Note that some biomarkers appear in different variations (e.g., ANCA (PANCA, PERINUCLEAR ANTI-NEUTROPHIL CYTOPLASMIC ANTIBODY; ANCA, ANTI-NEUTROPHIL CYTOPLASMIC ANTIBODY) and ASCA (ASCA, ANTI-SACCHAROMYCES CEREVISIAE ANTIBODY; ASCA-A, ANTI-SACCHAROMYCES CEREVISIAE ANTIBODY-IMMUNOGLOBULIN; ASCA-G, ANTI-SACCHAROMYCES CEREVISIAE ANTIBODY-IMMUNOGLOBULIN G)). (**C**) Number of patent families disclosing companion diagnostics biomarkers for IBD treatments. (**D**) Registered IBD clinical trials mentioning the respective endpoints (data regarding clinical trials on IBD downloaded from ClinicalTrials.gov on 15 December 2017; text search for different endpoints in trial title, description, and endpoint fields; trials classified as UC (512), CD (695), or both (191) were considered).

Classical diagnostic tools provide a snapshot of a few aspects of a very complex picture; the low sensitivity and variability of currently employed biomarkers, such as fecal calprotectin and CRP, limit their adoption in the clinic [25,26]. The current patent landscape for IBD biomarkers illustrates the current state of the field (Figure 2). The published patent families covering IBD biomarkers emphasize their diagnostic relevance for disease type identification (e.g., CD versus UC), determination of genetic predisposition, determination of inflammation/disease activity, and as companion diagnostics (Figure 2A). Notably, the top 20 biomarkers (on the basis of the number of patents) demonstrate the emphasis on a few common target categories; in addition to fecal calprotectin, these include anti–*Saccharomyces cerevisiae* antibodies (ASCA) and perinuclear anti-neutrophil cytoplasmic antibodies (p-ANCA) (Figure 2B). Especially relevant in terms of novel targeted treatment approaches, several companion diagnostics have been patented, including those for anti-TNF therapy and the specific anti-TNF drugs infliximab, adalimumab, and certolizumab (Figure 2C) [40].

Notably, the limited use of molecular biomarkers for diagnosis, stratification, and monitoring of IBD is also apparent in the available clinical trials data (Figure 2D). Complementing endoscopy and common symptom-quantifying research tools (e.g., the Crohn’s Disease Activity Index (CDAI) and Mayo score), calprotectin, and CRP are commonly used as molecular biomarkers in both CD and UC clinical trials. However, other evaluated biomarkers (ASCA/ANCA, lactoferrin, and Neutrophil gelatinase-associated lipocalin (NGAL)/Lipocalin 2 (LCN2)) have been limited to sporadic inclusion in these trials.

Overall, despite the use of some molecular biomarkers in clinical practice, there is a clear gap in terms of translating biomarker discoveries into clinical application [41]. This can be partially explained by the time required to translate omics discoveries from bench to bedside; it is also related to the complexity of designing reliable cross-border methods to ensure consistent results. Resolving this challenge and implementing individual omics profiling could enable a deeper understanding of the different molecular and clinical subtypes, thus facilitating easier and more precise diagnosis and personalized IBD treatment [42,43,44]. 

## 3. Applying Omics Analyses for IBD Research

Systems-level insights into disease mechanisms, the discovery of complex biomarkers, and, eventually, personal omics profiling are enabled by rapid advancements in molecular measurement technologies. Notably, novel sequencing technologies now facilitate the analysis of genome variations and transcriptome responses at a depth that was inconceivable just a few years ago [45]. In the IBD context, these novel sequencing methods have already provided us with novel insights, such as candidate biomarkers for CD [46]. However, as discussed, any complex biological disease, such as IBD, affects the system simultaneously on multiple levels; thus, multiple complementary analysis approaches are required to unravel relevant pathomechanisms and to identify robust biomarkers. 

**Figure 3:**
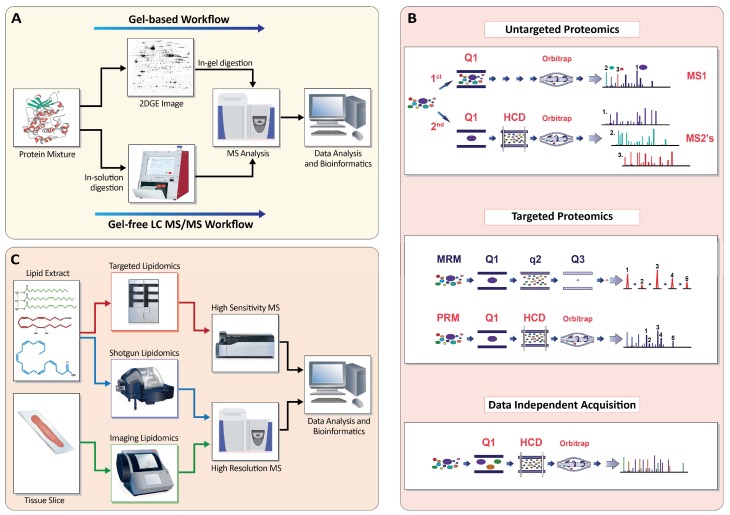
Schematic overview of the main proteomics and lipidomics technologies. (**A**) Gel-based and gel-free proteomics analysis workflows. In two-dimensional polyacrylamide gel electrophoresis (2D-PAGE), proteins are separated in two dimensions by molecular weight and isoelectric point. Proteins are then identified by MS after in-gel digestion. In LC-MS/MS approaches, proteins are digested into peptides prior to chromatographic separation and identification/quantification by MS. (**B**) Different MS-based proteomics approaches rely on distinct combinations of mass analyzers, such as quadrupoles (Q1/Q3) and high-resolution Orbitrap analyzers. To support identification, peptides can be fragmented in dedicated quadrupoles (q2) or higher-energy C-trap dissociation (HCD) cells. Untargeted proteomics involves two levels of mass spectra: the first (MS1) spectrum represents the masses of the unfragmented peptide ions. From this spectrum, peptide ions are selected for fragmentation in the HCD cell before analysis in the Orbitrap; the fragment ions are then represented in the second (MS2) spectrum. For targeted analyses, multiple reaction monitoring (MRM) commonly relies on a triple-quadrupole MS instrument. Specific peptide/fragment mass pairs (transitions) are selected and generated with quadrupole mass filters (Q1–Q3). During a targeted experiment, the mass spectrometer can cycle though several transitions to allow for multiplexing. Parallel reaction monitoring is a related technology that relies on a high-resolution fragment mass analyzer, such as an Orbitrap, rather than a quadrupole, allowing for simultaneous quantification of all fragment ions. In data-independent acquisition, ions are selected in bins over the entire analyzed mass range (Q1), all ions that fall into a mass bin are fragmented together (HCD), and the combined fragmentation spectra are deconvoluted during data processing. (**C**) Lipidomics can be divided into three main technologies, known as targeted, shotgun, and imaging lipidomics. Targeted and shotgun lipidomics are based, respectively, on the analysis of extracted samples, either by applying chromatographic separation or directly without separation before MS. Targeted lipidomics is performed primarily on instruments providing high sensitivity, such as triple-quadrupole MS, whereas shotgun lipidomics is preferably, but not exclusively, performed on high-resolution instruments, such as Orbitrap MS. Imaging lipidomics is based on the analysis of tissue slices through ionization of their surface molecules. The measured lipid ions are recorded by the mass spectrometer, then identified and quantified via dedicated software. For imaging lipidomics, molecules are localized onto the analyzed tissue. Figure elements based on Titz et al. [48]. See Appendix A for more details.

Here, we focus specifically on the question of how comprehensive analyses of proteome and lipidome alterations can effectively contribute to IBD research. Proteomics and lipidomics occupy different positions in the spectrum of omics methods: proteomics is the more mature and readily available method, whereas lipidomics allows completely new insights into the structure–function links of this important class of molecules. Notably, both omics methodologies, driven especially by improved MS instrumentation, are evolving rapidly, preparing the ground for future discoveries. In the following sections, we briefly summarize the contributions of these omics technologies to the investigation of IBD pathomechanisms and the identification of IBD biomarkers, before highlighting key considerations and possible improvements for future studies. As technical background, we provide a concise technical summary of current proteomics and lipidomics methods in Figure 3 and the Appendix A, with additional details available in recent review articles [31,47,48,49,50]. 

### 3.1. Mechanistic Insights Gained Using Proteomics and Lipidomics

Proteomics and, to a lesser extent, lipidomics have already been used successfully to investigate IBD pathomechanisms, including the inflammatory response, epithelial barrier function, and gut microbiome (Table S1). 

The molecular coverage and quantification accuracy of current proteomics and lipidomics approaches now enable investigation of the complex immune response directly in IBD patient samples. For example, a recent proteomics investigation further elucidated the innate immune response in IBD [51]: 5711 proteins were identified in mucosal colon biopsies without visible surface inflammation. In total, 46 proteins differed in abundance between UC and control colon tissue; UC tissue exhibited clear enrichment of upregulated neutrophil and neutrophil extracellular trap proteins [52]. This identification of clear signs of chronic inflammation, even in the absence of visible surface inflammation, highlighted the need for further studies into the chronic inflammation state in IBD patients [53]. 

For lipidomics, a recent case–control study demonstrated how this omics method can be applied to investigate disease states in mucosa samples [54]: inflamed mucosa showed increased levels of seven eicosanoids (prostaglandin (PG) E2, PGD2, thromboxane B2, 5-hydroxyeicosatetraenoic acid (HETE), 11-HETE, 12-HETE, and 15-HETE) that correlated with the degree of inflammation [54].

Both proteomics and lipidomics have also been employed to investigate IBD-relevant immune-cell responses. Studies have implicated Th17 T-cells in IBD pathogenesis, particularly in CD-associated dysregulation of immune responses [55,56]. Riaz et al. compared the proteomes of human Th1 and Th1/Th17 clones derived from intestinal biopsies of CD patients [57]. In total, 7401 proteins have been quantified; 334 were differentially expressed between Th1 and Th1/Th17 clones. Consistent with their functions in immune responses, cytotoxic proteins, such as granzyme B and perforin, were more abundant in Th1 than in Th17 cells. However, only a subgroup of Th1 cell clones from CD patients, characterized as CD28-positive and natural killer group 2 member D-negative, expressed these cytotoxic features; this suggested a larger-than-expected diversity in the T cell-mediated immune response in CD. In another recent example, proteomics of regulatory T-cells (CD4^+^Foxp3^+^) (Treg) led to the identification of a protein, THEMIS, as a checkpoint control in the suppressive function of Treg cells [58]. In a related context, lipidomics pinpointed specific differences in the response of macrophages from CD patients upon activation with heat-inactivated *Escherichia coli*, with significantly lower levels of newly synthesized phosphatidylinositol 16:0–18:1 [59].

As discussed earlier in this review, the disruption of intestinal epithelium integrity is an early event in IBD pathogenesis. Proteomics has been used to study differences between intestinal epithelial cells isolated from IBD patients and controls, leading to the identification of activated cellular stress responses in IBD [60,61]. 

Given the relevance of intestinal mucus as an intestinal barrier, lipidomics was used to investigate the phosphatidylcholine (PC) lipidome profile of rectal mucus obtained from IBD patients and control subjects [62,63]. UC patients displayed significantly lower levels of PC and lyso-PC compared with CD patients and controls. Interestingly, treatment of UC patients with a special formulation of PC, which exhibited delayed release in the gut, showed clinical efficacy [64], resulting in an improvement of the clinical activity index [65].

Genomics methods are at the forefront of the dissection of gut microbial populations. Nevertheless, metaproteomics is emerging as an effective approach to gain further insights into the complex interactions between the gut microbiome and host environment in health and disease [66]. In a recent study, a systems biology approach, including proteomics, was used to further understand the host–microbe cross-talk in new-onset CD [67]. The authors analyzed microbiota of the mucosa-luminal interface by 16S rDNA gene sequencing and combined this with a quantitative proteomics investigation of the host proteomes in mucosal biopsies; 320 of 3323 quantified proteins were significantly differentially expressed, including several mitochondrial proteins that were significantly downregulated in CD patients compared with controls. Interestingly, this included several proteins involved in H_2_S detoxification. Concomitantly, metagenomics data showed an increase in the relative abundance of microbial H_2_S producers, indicating *Atopobium parvulum* as a central hub. Finally, supporting a possible causal link, in a follow-up experiment with IL-10-deficient mice, *A. parvulum* was shown to induce colitis, which was mitigated by an H_2_S scavenger [67]. 

### 3.2. Biomarker Identification Using Proteomics and Lipidomics

In addition to these mechanistic studies, proteomics and lipidomics have been applied in several IBD biomarker identification studies (Table S1). In total, we identified 18 published studies that were performed specifically to identify biomarkers. Only a single study employed lipidomics, and most recent proteomics studies tended to employ liquid chromatography coupled to tandem MS (LC-MS/MS) rather than 2D-PAGE technologies, reflecting a shift to more recent and more potent proteomics technologies. Most of the studies (11 in total) focused on blood/serum as the sample source; five studies focused on colon samples, two studies on feces, and a single study on saliva, aligned with the convenience of sampling (serum, feces, and saliva) and proximity of the sampling site to the disease process (colon biopsies and feces). These studies were undertaken to identify biomarkers in at least three different application areas: diagnosis, patient stratification, and treatment selection and response monitoring. 

For example, a recent study, which was focused on IBD diagnosis, combined discovery proteomics with targeted verification experiments to identify markers for intestinal complications of CD (untreated/balloon-dilated strictures and non-healed abscesses/fistulas) [68]. The derived serology panel was able to indicate complications in CD with 70% sensitivity and 72.5% specificity. In a lipidomics study, plasma lipid profiles were compared across healthy controls and UC and CD cases [69]; this resulted in the identification of 33 lipid species, primarily belonging to ether-lipids, that were significantly correlated with CD.

Another recent proteomics study focused on proteins at the mucosal-luminal interface to identify biomarker candidates for pediatric IBD-associated colitis [70]. Two distinct four-protein panels were identified that were able to discriminate active IBD from non-IBD and pancolitis from non-pancolitis, with reported high sensitivity and specificity (>0.95). However, these estimates were obtained from the discovery cohort, not from an independent validation cohort; thus, it is unclear how these panels might perform with real clinical samples. Nevertheless, one protein from each panel, LTA4H, and catalase, was also measured in stool samples from an independent cohort, demonstrating elevation in IBD versus non-IBD stool samples. These data support the selection of IBD-relevant proteins and demonstrate the possibility that these markers can be measured in more easily accessible stool samples.

The identification of specific IBD phenotypes in patients diagnosed with indeterminate colitis (IC) has been implemented recently by using disease stratification biomarkers. Among young (<18-year-old) IBD patients, 15−30% cannot be fully classified and are therefore diagnosed with IC. While individual serum cytokine profiles are unable to distinguish among IBD subtypes [71,72], both transcriptomics [71] and MALDI imaging proteomics profiles of colon biopsies [73,74] showed clear differences between UC and CD, supporting the feasibility of the development of biopsy biomarkers for disease stratification. 

Another study, intended to improve disease stratification, identified protein markers that were able to discriminate between CD and UC in children with new-onset IBD [75]. Two candidate biomarker panels were established: a five-protein panel able to discriminate IBD from control cases with 95.9% accuracy, and a 12-protein panel able to discriminate between CD and UC patients with 80% accuracy in the validation cohort.

Proteomics has also been employed to identify treatment response biomarkers for IBD. Anti-TNF therapy with infliximab is now commonly used to control inflammation in IBD patients [76]. However, the factors predicting response, as well as the cellular mechanisms leading to the therapeutic response, are not fully understood. In a population of 43 UC patients, Magnusson et al. investigated infliximab treatment response in blood and inflamed colon biopsies [77]. Treatment response was associated with lower levels of macrophage markers CD14 and CD86 and the chemokine CCL2. In addition, proteomics identified reduced levels of tenascin-C (a glycoprotein of the extracellular matrix) in biopsies upon treatment, which was consistent with reduced tenascin-C levels in serum. 

Finally, another study investigated differences in the serum protein profiles of children with IBD before and after treatment with infliximab or corticosteroids [78]. In total, 18 proteins differed significantly in both treatment groups with the same directionality; consistently downregulated proteins were associated with inflammatory processes. 

## 4. Proteomics and Lipidomics in IBD Research—Going Forward

**Figure 4:**
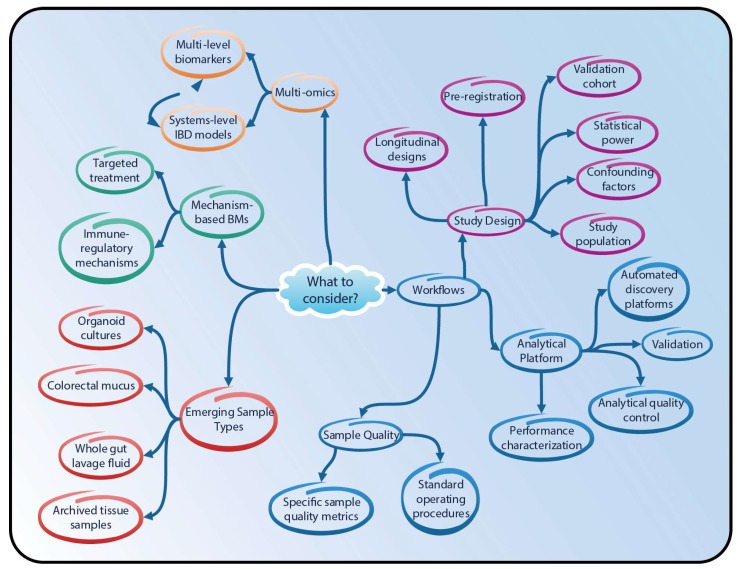
Aspects to consider in the design and conduct of IBD biomarker studies.

As illustrated in the above sections, both proteomics and lipidomics can provide further insights into IBD pathomechanisms and support the development of novel biomarker assays. However, thus far, both these omics technologies have not exploited their full potential. The published studies provide a fairly heterogeneous picture of the field, with interesting case studies; however, there is a lack of clear direction regarding how these findings can be further developed and, eventually, translated into clinical practice. This is especially evident in the biomarker discovery field, where only a few studies have included a validation cohort, and there is an obvious disparity between reported biomarker candidates and those used in the clinic. Clearly, translating novel findings is a long process. Nevertheless, design of new studies can clearly benefit from the newly generated data taking into consideration some key aspects (Figure 4).

**Omics technologies.** MS-based technologies for the quantification of proteome and lipidome changes are evolving rapidly (see Appendix A). The molecular coverage and sample throughput available today were unimaginable just a few years ago. Noteworthy, for example, is the emergence of so-called data-independent acquisition approaches, which now enable very comprehensive and reproducible snapshots of the proteome in large sample cohorts [79]. This growth will likely continue, and future studies will benefit from advances in omics technologies. 

**Sample quality.** However, it is important not only to focus on the specific omics technology employed in a given situation but also to design the entire sample collection, processing, and analysis workflow from a robustness and quality perspective. Sample quality is among the most important aspects required for a successful biomarker study [80]. Well-defined standard operating procedures for sample collection and handling can ensure high-quality, reproducible sampling [81], as reported for serum/plasma [82] and cerebrospinal fluid [83]. 

**Analytical platform.** To ensure the overall quality of the analytical platform, its performance must be characterized in depth (e.g., in terms of measurement accuracy and reproducibility). For proteomic studies, the scientific community has developed and published useful guidelines [84,85,86]. Rigorous analytical quality control is crucial and can be supported by automated LC-MS/MS performance monitoring systems [87,88], together with specific sample quality metrics (e.g., [89,90]). To prevent late failures of biomarker candidates, it has been suggested that a core method validation (e.g., including estimates of accuracy, precision, and limits of quantification) should be performed when designing biomarker discovery workflows [91]. Clearly, full method validation becomes essential when a newly discovered biomarker is moved toward clinical application. For this, recognized validation guidelines are to be followed, such as those provided by the Clinical and Laboratory Standards Institute (e.g., [92]) and those recognized by the U.S. Food and Drug Administration [93]. The Eurachem guide by Magnusson and Örnemark is a useful reference for general analytical method validation guidelines [94]. Moreover, especially for large-scale studies, automation can be considered to make biomarker discovery platforms more robust, as recently demonstrated by several groups (Table 1). 

**Study design.** Ultimately, especially for biomarker studies, the study design will determine the success of a project. Relevant considerations for the design of biomarker identification and validation studies have been outlined [81,98,99]; these include clearly defined inclusion and exclusion criteria, control of confounding factors, and well-defined statistical analysis plans with statistical power calculations [100,101]. If possible, longitudinal designs (i.e., following biomarkers in a subject over time) should be considered, as these are especially powerful for assigning the observed variability in a candidate biomarker to a biological response of interest, rather than to complex differences in the human population. Most importantly, however, no biomarker identification study should be planned without an independent study cohort to validate the findings. This is especially relevant considering that only 23% of the published plasma proteomics studies for biomarker discovery have been independently verified [91].

**Emerging sample types.** Another generally important aspect is the selection of the biological source material. A broad spectrum of biological sample types, ranging from serum to colon biopsies and feces, has been leveraged for IBD investigations. Alternative sources could be considered to facilitate new discoveries. For example, preserved tissue samples stored in large biobanks could provide an alternative and, possibly, complement studies that use fresh tissue biopsies. Several groups have shown that archived tissue samples are suitable for proteomics studies [102,103,104,105], with a recent study applying proteomics to fixed colon mucosa samples [106]. Another promising sample type for IBD research is whole gut lavage fluid (WGLF) [107], which can be collected when the gastrointestinal tract is cleansed before colonoscopy or colon surgery. For example, in one study, lactoferrin was increased in the WGLF of IBD patients—it was thus proposed as a marker for intestinal inflammation [108]—while elevated levels of IL-1β were identified in the WGLF of CD patients with a high risk of relapse [109]. Finally, colorectal mucus could be further explored as a potential sample source. Previous studies have established that colorectal mucus is a rich source of potential IBD biomarkers, as demonstrated by the quantification of calprotectin and eosinophil-derived neurotoxin in mucus samples from IBD patients [110,111]. 

**Multi-omics.** Returning to the main theme, another key general consideration is which molecule class to profile. While we focus here on proteome and lipidome profiling methods, clearly, transcriptome, (epi-) genome, and metabolome profiling methods can be expected to yield very relevant insights. Importantly, IBD pathogenesis results from disturbances in the complex interplay of multiple biological processes; a comprehensive understanding of these processes will ultimately require integrative approaches combining several omics and non-omics endpoints within a systems biology framework [20,44,112,113]. Notably, Danese et al. recently emphasized the need for multi-omics integration to achieve comprehensive and effective IBD disease risk assessment, early and accurate diagnosis, effective targeted treatments, identification of the biological basis of disease states, and, finally, disease prevention [114]. Similarly, as mentioned, it has been argued that the overwhelming complexity of IBD can only be addressed with an integrative view of all pathological components, known as the “IBD interactome” [44]. Generating relevant and interpretable views of multi-omics data sets remains a challenge; however, several omics data integration methods are readily available [115], and novel methods are emerging (e.g., Multi-Omics Factor Analysis [116]).

The multi-level insights thus gained into IBD pathogenesis could then lead to the development of effective biomarkers that are specific for central regulatory nodes in the disease process. Importantly, how feasible are such multi-level or multi-omics biomarkers? The benefits of such integrative biomarker panels have already been recognized for IBD, with commercially available products that combine serologic, genetic, and inflammation markers to support IBD diagnosis (Table S2).

## 5. Conclusions

IBD is a complex disease with a multifactorial etiology that is affected by genetics, gut microbiota, immune response, and the environment [17,18,19]. Global molecular profiling and quantitative technologies, including proteomics and lipidomics—in combination with sophisticated computational analysis and integration approaches—are emerging as new tools able to generate high-throughput data, instrumental for further understanding of IBD pathogenesis and biomarker identification. 

**Figure 5:**
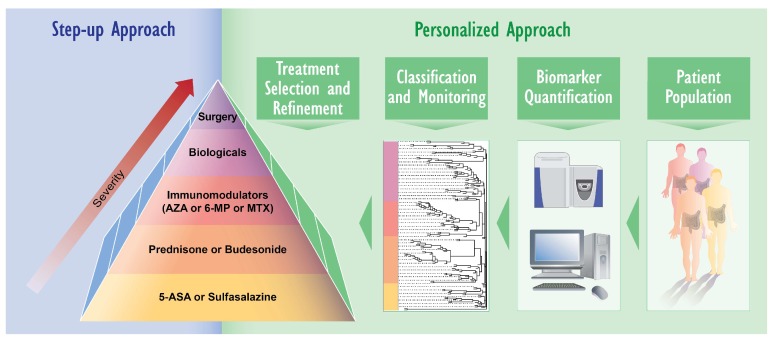
Step-up and personalized treatment approaches for IBD. In the classical “step-up” approach to IBD treatment, treatments are selected and aligned with disease severity, from the bottom to the top of the pyramid. The proteomics and lipidomics approaches discussed in this review will contribute to the development of personalized treatment of IBD patients. The discovery of predictive biomarkers will support the matching of personalized treatment, continuous monitoring of treatment responses, and further treatment refinements.

In this review, we have summarized the state-of-the-art proteomic and lipidomic approaches to IBD studies. The intrinsic conundrum of intestinal physiology and its derangement in gut pathologies put in place different players in IBD onset and development, thus creating different layers of complexity. The application of targeted omics studies will enable unraveling of the contributions of each tissue subcomponent. The rapid evolution of these technology platforms will transform the current step-up approach [117], in which treatment is chosen on the basis of disease severity and treatment responsiveness. Importantly, it will accelerate biomarker discovery, indicate novel therapeutic avenues for the clinical management of IBD, and facilitate the possibility of continuous monitoring of the treatment response of individual patients, allowing further refinement of treatment. Implementing the output of omics techniques, in combination with (epi-)genomics, microbiome studies, and nutrigenomics, will eventually lead to personalized medicine for IBD patients (Figure 5). 

## Figures and Tables

**Table 1:** Recently published LC-MS/MS-based protein biomarker identification workflows.

Quantification Approach	Abundant Protein Depletion	Separation	Scale(#Proteins/#Samples)	Precision	Comment	References
iTRAQ (discovery)	Yes	SCX C18	Pool of 20 samples		Biomarker panel for diabetic kidney disease developed	[95]
MRM (validation)	Yes	C18	8/572	CV ~8%		
Label-free	No	C18	~437/319	CV <20% for 71% of proteins	Applied within weight-loss study	[96]
TMT	Yes	C18	~190/~1000	Average CV of 12% for internal standard	Applied within multicentered dietary interventionstudy	[97]

Coefficient of variation (CV); strong cation exchange chromatography (SCX); octadecyl carbon chain chromatography (C18).

## Supplementary Materials

Supplementary Materials can be found at http://www.mdpi.com/1422-0067/19/9/2775/s1. Table S1. Proteomics and lipidomics studies for IBD. Table S2. IBD biomarker products. References [51,54,57,58,59,60,61,62,67,68,69,70,73,74,75,77,78] are cited in the supplementary materials.

## Abbreviations

IBD	inflammatory bowel disease
MS	mass-spectrometry
UC	ulcerative colitis
CD	Crohn’s disease

## Appendix A

This appendix provides additional details on common proteomics and lipidomics methods.

### Appendix A.1. Profiling the Proteome

#### Appendix A.1.1. Gel-Based Approaches (2D-PAGE)

Two-dimensional polyacrylamide gel electrophoresis (2D-PAGE) is the classical approach for differential proteome analysis (Figure 3A). Proteins are separated in two dimensions on a gel, based on their isoelectric point and molecular mass, allowing for quantification of up to 2000 proteins on a single gel. A variant of 2D-PAGE is difference gel electrophoresis, in which proteins of different samples are labeled with different fluorescent dyes, allowing for multiplexed analyses [118,119]. Disadvantages of 2D-PAGE include the high protein amount needed per sample (at least 150 µg) and the lower sensitivity of detection of biological effects compared to gel-free approaches [120,121].

#### Appendix A.1.2. Gel-Free Approaches (LC-MS/MS)

Besides gel-based approaches, liquid chromatography (LC) is the most commonly used method in the proteomics field for sample fractionation before mass spectrometry (MS) (Figure 2A,B). Bottom-up workflows are currently most common. In these workflows, proteins are first cleaved/digested into peptides by specific proteases, such as trypsin. Subsequently, sample fractionation, identification, and quantification are performed on the level of the derived peptides. In contrast, in top-down workflows, intact (non-digested) proteins are analyzed directly. For fractionation, the LC approach takes advantage of differences in the physiochemical properties of proteins and peptides (i.e., their size, charge, and hydrophobicity). LC coupled to tandem MS (LC-MS/MS) has brought about many advances, including increased sensitivity and improved speed, with the ability to produce high-throughput datasets. Peptides in the femtomolar (10^−15^) to attomolar (10^−18^) range can be detected in tissues and biological matrices with a mass accuracy of <10 ppm [31,122].

Different quantification methods can be used with bottom-up LC-MS/MS approaches. Label-free quantitative (LFQ) techniques directly estimate the (relative) concentrations of unmodified proteins. Spectral-counting is a classical LFQ technique that estimates protein abundances based on the number of observed MS spectra for a given protein. However, LFQ techniques that estimate protein abundances based on the integrated MS signal intensities of their peptides produce much more accurate estimates [123]. LFQ allows comparison of multiple samples, covers a broad concentration range, and does not require further sample pretreatment. However, compared with the label-based methods discussed below, LFQ generates more variable protein abundance estimates and does not allow for multiplexed analysis of several samples in a single MS experiment [124,125].

In label-based quantification methods, samples are specifically modified before or during analysis. The stable isotope labeling with amino acids in cell culture (SILAC) approach relies on the metabolic (in vivo) incorporation of specific “light” or “heavy” isotope forms of selected amino acids (most commonly arginine and lysine) into proteins [126,127,128]. Differentially labeled samples are mixed and analyzed together, and for relative quantification, the areas under the elution peaks from the “light” and “heavy” labeled versions of each detected peptide are compared directly [126].

Alternatively, in chemical labeling approaches, peptide mixtures derived from the protein digestion step are tagged with specific chemical labels for each sample that is to be compared. Examples are the closely related isobaric tag for relative and absolute quantitation (iTRAQ^®^) and the tandem mass tags (TMT^™^) approaches, in which specific chemical tags are attached to all peptides in a protein digest via free amines at the peptide N-terminus and lysine side chains. Labeled samples are then pooled and analyzed simultaneously. The main advantages of isobaric tag-based quantification are the simultaneous comparison of large numbers of samples (up to 10 for TMT), reduction of required MS runs (and thus total analysis time), as samples are pooled before MS analysis, and the low probability of introducing experimental errors during analysis due to the pooling of all the samples to be analyzed [48]. The limitations of the technique are the low dynamic range and the requirement for the protein profiles to be similar.

#### Appendix A.1.3. Targeted MS

As the need for accurate quantification of a specified set of peptides/proteins across multiple samples has grown, targeted approaches have been developed for biomarker quantification. Multiple reaction monitoring (MRM) and parallel reaction monitoring (PRM) are the main MS-based targeted quantification methods (Figure 3B). In both approaches, quantification is performed on the peptide level, quantifying selected, surrogate peptides derived from the enzymatic digestion of the proteins of interest. Commonly, corresponding heavy isotope-labeled peptides are included as internal standards to allow for robust and absolute quantification. These methods are mostly suitable for quantification of tens to hundreds of targeted proteins in complex matrices with attomole-level limits of detection [129]. This makes these targeted methods especially useful for profiling well-defined target panels [130] and validation of results from untargeted proteomics experiments, including biomarker validation (e.g., [68]).

#### Appendix A.1.4. Data-Independent Acquisition

In addition to multiplexed PRM/MRM approaches, data-independent acquisition (DIA) is among the most rapidly evolving methods in the proteomics field. DIA methods can be regarded as the middle ground between untargeted and targeted proteomics approaches, as they combine untargeted MS acquisition with targeted computational analyses (Figure 3B) [131]. In DIA approaches, the mass spectrometer is programmed to acquire peptide masses and their fragmentation patterns systematically and comprehensively across the whole proteome. Specific spectral libraries—as well as specific database search algorithms—are then used to unravel these complex fragmentation datasets and to derive peptide and protein expression profiles. DIA methods are characterized by high quantification accuracy with broad proteome coverage, a low number of missing values, and the possibility of conducting targeted re-analyses of a data set [131,132].

#### Appendix A.1.5. Beyond Expression Changes: Post-Translational Modifications

MS-based proteomics methods are also especially well-suited to investigate the role of post-translational modifications (PTM) of proteins. PTMs are important for regulation of cellular processes, including cellular localization of protein, regulation of protein function, and protein complex formation. These covalent protein modifications occur during or after protein biosynthesis. MS/MS provides a series of analytical features that enable the facile characterization of modified proteins via amino acid sequencing and specific detection of post-translationally modified amino acid residues [133]. Two commonly studied PTMs are phosphorylation [134] and glycosylation [135]. Protein phosphorylation is critically important for signaling cascades (e.g., those involved in immune-regulatory interactions). With the importance of glycosylated proteins, such as mucins, in IBD, studies of the glycoproteome can be also expected to yield relevant insights [136] and possibly data related to how glycosylation affects the microbial–host interactions in IBD [135].

#### Appendix A.1.6. Non-MS-Based Proteomics Methods

Other proteomics methods are based on antibodies or alternative protein binders. For example, Luminex multi-analyte profiling (xMAP^®^) technology is a bead-based, multiplexed immunoassay (i.e., antibody-based) system in a microplate format. The system is able to detect up to 500 targets in a single sample simultaneously, depending on system design [137]. Advantages of the xMAP^®^ technology are its high-throughput format, accessibility via a broad and expanding number of immunoassay kits, and economy via a significant reduction in time and costs compared with running multiple Western blots or enzyme-linked immunosorbent assays (ELISA) [137].

Reverse-phase protein microarrays (RPPA) are an emerging alternative technology for the quantification of (phospho-) protein panels. RPPAs use spotted lysate microarrays, which are subsequently probed by different validated antibodies to detect protein expression changes. Advantages of RPPAs are cost-effectiveness, because the system involves multiplexed analysis of more than 1000 samples on a single slide, and high sensitivity (ng of protein lysates spotted, detection of attomoles of a specific protein).

Finally, alternative protein binders can be used effectively for quantification. Slow Off-Rate Modified Aptamer technology (SOMAmer^®^) is a new, aptamer-based, proteomic technology for biomarker discovery. It uses a new generation of aptamers that contain chemically modified nucleotides. Proteins present in complex matrices, such as plasma, are measured in a process that transforms a signature of protein concentrations into a corresponding signature of DNA aptamer concentrations, which is quantified on a DNA microarray. This technology allows for the measurement of thousands of proteins from small sample volumes [138].

### Appendix A.2. Profiling the Lipidome

#### Appendix A.2.1. Shotgun Lipidomics

As for proteomics, current lipidomics methods are mostly MS-based. In shotgun lipidomics approaches, a crude lipid extract is injected directly into the MS instrument (Figure 3C). This method simplifies the setup, reduces the requirement for additional quality controls, and is highly quantitative. With this approach, hundreds of lipid species can be identified and quantified precisely from a small sample size (e.g., 1 µL of plasma) [139,140].

#### Appendix A.2.2. Targeted Lipidomics

In targeted lipidomics, LC is combined with direct MS detection (Figure 3C). Like in targeted proteomics, MRM and PRM approaches are commonly applied [141]. This allows analysis of low-abundance lipid species, complex lipid mixtures that cannot be detected using shotgun approaches, and ensures accurate quantification of specific lipid species. Sensitivity and selectivity are gained by separating both analytes and impurities, permitting precise detection of low abundance lipids.

#### Appendix A.2.3. FLUX Lipidomics

Similar to SILAC for proteomics, stable isotope labeling of endogenous lipids can be performed to measure the turnover and dynamics of lipid species. This technology is referred to as FLUX lipidomics, or stable isotope-labeled tracers, and is based on stable isotopes (e.g., deuterium, ^13^C, ^15^N) in various lipid metabolic substrates (e.g., ^13^C-labeled fatty acids). The labeled tracer will introduce a mass shift in the incorporated lipid product that can be distinguished readily by MS. Thus, when delivered to cells, the incorporation rate of the stable isotope into lipid species can be quantified using shotgun and/or targeted lipidomics. This permits the precise determination of lipid species turnover in cells [142]. FLUX lipidomics comprehensively resolves lipid turnover and dynamic metabolic flow of the global lipid metabolism in biological systems.

#### Appendix A.2.4. Imaging Lipidomics

Using the imaging mass spectrometry (IMS) approach, MS can also spatially resolve the distribution of lipids on a tissue slide (Figure 3C). Knowing how lipid species are distributed in tissues can help understand lipid metabolism, disease states (e.g., by comparing lipids between healthy and diseased tissue regions), and biological processes. IMS is typically performed on fresh or frozen tissues using slices (<20 μm thickness) placed onto special supports or coated glass microscope slides [140]. Matrix-assisted laser desorption/ionization (MALDI) is used to generate lipid ions for spatially resolved MS analysis from these tissue slices. Current technologies permit spatial resolution down to 5 µm, providing high precision in the localization of the recorded ions [143].
