# Supplementary material for: Proteomics and Lipidomics in Inflammatory Bowel Disease Research: From Mechanistic Insights to Biomarker Identification
Source: Int J Mol Sci. 2018 Sep 15;19(9):2775. doi: 10.3390/ijms19092775 (PMC6163330; doi:10.3390/ijms19092775)
Supplement: Supplementary file 1 [file ijms-19-02775-s001.pdf]

Review

Proteomics and Lipidomics in Inflammatory Bowel Disease Research: From Mechanistic Insights to Biomarker Identification

Bjoern Titz[1,*,†], Raffaella M Gadaleta [1,†], Giuseppe Lo Sasso [1,†], Ashraf Elamin [1], Kim Ekroos [2], Nikolai V Ivanov [1], Manuel C Peitsch [1] and Julia Hoeng [1,*]

[1] PMI R&D, Philip Morris Products S.A., Quai Jeanrenaud 5, CH-2000 Neuchatel, Switzerland; RaffaellaMaria.Gadaleta@pmi.com (R.M.G.); Giuseppe.LoSasso@pmi.com (G.L.S.); Ashraf.Elamin@pmi.com (A.E.); Nikolai.Ivanov@pmi.com (N.V.I.); Manuel.Peitsch@pmi.com (M.C.P.)

[2] Lipidomics Consulting Ltd., Irisviksvägen 31D, 02230 Esbo, Finland; kim@lipidomicsconsulting.com

* Correspondence: bjorn.titz@pmi.com (B.T.); julia.hoeng@pmi.com (J.H.); Tel.: +41-(58)-242-2312 (B.T.); +41-(58)-242-2892 (J.H.)

† These authors contributed equally to this work.

Supplementary Tables

Supplementary Table S1. Proteomics and lipidomics studies for IBD. Studies identified via a PubMed search [("inflammatory bowel disease" OR "Crohn's disease" OR "ulcerative colitis") AND (LIPIDOMICS OR PROTEOMICS)] and complemented with Google Scholar search results.

Category[1]	Species	Sample Type	Groups[2]	Technology	Main Findings	Reference
**BM**	Human	Colonic mucosa biopsies	Discovery: CD (15), UC (15), HC (20) Validation: CD (15), UC (15), HC (19)	Proteomics LC-MS/MS	Five-protein panel able to discriminate IBD versus control cases with 95.9% accuracy, and a 12-protein panel discriminating between CD and UC patients with 80% accuracy in the validation cohort.	[1]
**BM**	Human	Blood/Serum	UC (30) HC (30)	Proteomics 2D-PAGE	Twelve serum proteins differed between UC and HC	[2]
**BM**	Human	Blood/Serum	UC (24) HC	Proteomics 2D-PAGE	Upregulation of ceruloplasmin and apolipoprotein B-100 specifically in children	[3]
**BM**	Human	Blood/Serum	Stricturing CD (9) Nonstricturing CD (9) UC (9)	Proteomics LC-MS/MS	Stratification of IBD patient subgroups	[4]
**BM**	Human	Blood/Serum	CD UC HC	Proteomics LC-MS/MS	Peptides of the secreted phosphoprotein 24 (SPP24) differentiated IBD from controls	[5]
**BM**	Human	Blood/Serum	CD with and without intestinal complications	Proteomics LC-MS/MS	Serology panel of three proteins to identify CD with complications	[6]
**BM**	Human	Blood/Serum	UC CD HC	Lipidomics	Thirty-three lipids (primarily ether-lipids) negatively associated with CD	[7]
**BM**	Human	Colon biopsies	UC CD	Proteomics MALDI imaging	Clear differences between UC and CD for stratification, but specific molecules not identified	[8,9]
**BM**	Human	Colonic mucosa biopsies	UC (4) CD (3) HC (3)	Proteomics LC-MS/MS	Twelve upregulated proteins were shared between the UC and CD cases	[10]

			UC-related inflammatory polyps (2)			
**BM**	Human	Intestinal mucosal-luminal interface aspirates	18 non-IBD<br>42 IBD	Proteomics<br>LC-MS/MS	Two 4-protein panels that distinguished active IBD from non-IBD and pancolitis from non-pancolitis in UC, respectively (discovery cohort, limited validation for two proteins in stool samples)	[11]
**BM**	Human	Salivary exosomes	37 UC, 11 CD, 10 healthy individuals	Proteomics<br>LC-MS/MS	PSMA7 increased in UC and CD	[12]
**BM**	Mouse interleukin-$10^{-/-}$ model	Blood/Serum	Longitudinal during development of IBD-like disorder	Proteomics<br>2D-PAGE	Candidate markers for global, intestinal, and chronic inflammation	[13]
**BM**	Mouse G-alpha(i2)$^{-/-}$ IBD model	Fecal samples	Diseased, non-diseased (wild-type) mice	Proteomics<br>LC-MS/MS	Ten differentially abundant proteins, including higher levels of peptidase D in diseased mice	[14]
**BM, TR**	Human	Blood/Serum	CD with and without infliximab response (20)	Proteomics<br>2D-PAGE	Initial prediction model of treatment response, including platelet aggregation factor 4 (PF4)	[15]
**BM, TR**	Human	Blood/Serum	Infliximab responders in remission (6), responders (6), and non-responders (6) [16]	Proteomics<br>2D-PAGE	Candidate serum markers for treatment response	[16]
**BM, TR**	Human	Blood/Serum<br>Inflamed colon biopsies	Infliximab responders, non-responses	Proteomics<br>LC-MS/MS	Reduced tenascin-C level in biopsies and serum upon treatment	[17]
**BM, TR**	Human	Blood/Serum	Children with IBD, before and after treatment with corticosteroids (12) or infliximab (12)	Proteomics<br>SOMAmer	Five proteins demonstrated consistent downregulation upon treatment and were associated with inflammatory processes	[18]
**BM,DM**	Human	Colon biopsies	Acute UC<br>HC	Proteomics<br>2D-PAGE	Distinguish inflamed and non-inflamed samples, increase in energy metabolism and oxidative stress proteins in UC	[19]
**DM**	Human	Adenocarcinoma cell line<br>Purified colon epithelial cells	Cytokine treated cell line/isolated cells from UC, CD, and HC	Proteomics<br>2D-PAGE	Indoleamine-2,3-dioxygenase (IDO1) upregulation in UC/CD, linked to immune-tolerance	[20]
**DM**	Human	Blood/Macrophages	Stimulation with heat-inactivated *Escherichia coli*	Lipidomics	Alteration in lipid levels, including ceramides	[21]
**DM**	Human	Endoscopic sampling of microbiome by mucosal lavage	UC<br>CD<br>HC	Proteomics (targeted & untargeted)	Protein modules associated with intestinal location and disease state	[22]

**DM**	Human	Intestinal biopsies	Th1 and Th1/Th17 T-cell clones from CD	Proteomics LC-MS/MS	Only subgroup of Th1 expresses cytotoxic features	[23]
**DM**	Human	Isolated intestinal epithelial cells (inflamed and non-inflamed regions)	CD<br>UC<br>CRC (control)	Proteomics 2D-PAGE	Alterations in signal transduction, stress response, and energy metabolism in IBD	[24]
**DM**	Human	Isolated intestinal epithelial cells	CD<br>HC	Proteomics LC-MS/MS	Upregulation of protein folding and ubiquitin processes in CD	[25]
**DM**	Human	Lymphocytic cell line	IL-23 stimulation	Proteomics LC-MS/MS (phospho-proteomics)	STAT3 involvement, regulatory role of pyruvate kinase isozyme M2	[26]
**DM**	Human	Mucosa biopsies	Active UC<br>Inactive UC<br>Nonspecific colitis<br>HC	Proteomics 2D-PAGE/MS	Colonocyte mitochondrial dysfunction and perturbed mucosa immune regulation in the pathogenesis of UC	[27]
**DM**	Human	Mucosa biopsies (non-inflamed)	UC<br>HC	Proteomics LC-MS/MS	Role of neutrophil extracellular traps in UC	[28]
**DM**	Human	Mucosa biopsies	UC<br>HC	Lipidomics	Inflamed mucosa in UC with elevated levels of seven eicosanoids	[29]
**DM**	Human	Mucosa biopsies	UC	Proteomics LC-MS/MS (label-free)	168 differentially abundant proteins between UC and HC	[30]
**DM**	Human	Mucosa biopsies	CD<br>HC	Proteomics LC-MS	Downregulation of mitochondrial proteins in CD, including H_2S detoxification enzymes	[31]
**DM**	Human	Rectal mucus	UC<br>CD<br>HC	Lipidomics	Lower levels of PC and lyso-PC in UC	[32]
**DM**	Human (twin pairs)	Stool samples/Microbiome	CD<br>HC	Proteomics LC-MS	CD associated with alterations in bacterial carbohydrate metabolism and bacterial-host interactions; and increase in host proteins involved in epithelial integrity and function	[33]
**DM**	Human	Treg cells and conventional T lymphocytes ($CD4^+Foxp3^-$)	Isolated cell populations	Proteomics LC-MS/MS	Themis protein as a checkpoint control in the suppressive function of Treg cells	[34]
**DM**	Human cell line	Adenocarcinoma cell line	Cytokine treated<br>Untreated	Proteomics 2D-PAGE (ubiquitin staining)	Ubiquitin-mediated regulation of chaperones in inflammatory response	[35]

**DM**	Mouse TNF transgenic	Microbiome	IBD and control mice	Proteomics LC-MS	Metaproteomics complemented genomics methods for the analysis of microbial communities	[36]
**DM**	Mouse adoptive T-cell transfer model	Microbiome	IBD and control mice	Proteomics LC-MS (activity-based probes for enzyme classes)	Quantitative alterations in both host and microbial proteins due to intestinal inflammation	[37]
**DM**	Mouse	Serum	DSS-treatment & recovery period	Lipidomics	Involvement of lipid mediators (such as resolving D1) in intestinal healing process	[38]
**DM**	Primary murine bone marrow–derived macrophages	Cell culture	Wild-type and Atg16l1 (autophagy-related gene) knockout	Proteomics LC-MS/MS	Limited set of differentially abundant proteins upon Atg16l1 knockout, prominently sequestosome-like receptors	[39]
**GA**	Human	Serum	CD UC HC	Proteomics Antibody microarray	SNP in CD regulated levels of MST1 in serum	[40]
**GA**	Human	Serum	Diverse	Proteomics SOMAmer	Genome-proteome-disease sub-network that associated CD with four genomic loci (MST1, IL23R, IL18R1, and C7)	[41]
**TR**	Mouse Mdr1a$^{-/-}$ model	Colon	Polyphenol-treatment	Proteomics 2D-PAGE	Treatment decreased inflammatory and fibrinogenesis proteins and increased xenobiotic metabolism enzymes	[42]
**TR**	Mouse Mdr1a$^{-/-}$ model	Colon	Curcumin treatment	Proteomics 2D-PAGE	Treatment decreased inflammatory proteins and increased xenobiotic metabolism enzymes	[43]
**TR**	Mouse DSS-model	Colon	Celastrol treatment	Lipidomics	Celastrol treatment restored control-like lipid profiles	[44]

[1] Categories: disease mechanisms (DM); candidate biomarker identification (BM); treatment response characterization (TR); genotype association (GA). [2] Group abbreviations: healthy control (HC); colorectal carcinoma (CRC). Phosphatidyl-choline (PC).

Supplementary Table S2. IBD biomarker products.

PRODUCT	ASSIGNEE	MARKER(S)	MATRIX	APPLICATION	PATENT IDENTIFIED	FDA APPROVED
**ASCA-CHEK™**	TECHLAB INC.	ASCA	Feces	CD	Yes	Yes
**COLOGUARD**	EXACT SCIENCES	DNA mutation/methylation and hemoglobin	Feces	Colorectal cancer	Yes	Yes
**LACTOFERRIN CHEK®**	TECHLAB INC.	Lactoferrin	Feces	IBD & treatment response	Yes	Yes
**LACTOFERRIN EZ VUE®**	TECHLAB INC.	Lactoferrin	Feces	IBD	Yes	Yes
**LACTOFERRIN SCAN®**	TECHLAB INC.	Lactoferrin	Feces	IBD & treatment response	Yes	Yes
**PHICAL TEST**	CALPRO AS	Calprotectin	Feces	IBD	Yes	Yes
**CALPROLAB™ ELISA TEST (ALP/HRP)**	CALPRO AS	Calprotectin	Feces Blood Tissue fluids	IBD & treatment response	Yes	
**CALPROTECTIN ELISA**	EUROIMMUN	Calprotectin	Feces	IBD & treatment response		
**CROHN'S PROGNOSTIC**	PROMETHEUS LAB INC.	Antibodies and genetic markers	Serum	CD complications	Yes	
**DIBICOL**	INDEX PHARMACEUTICALS	Gene panel	Colon biopsy	IBD, UC vs. CD	Yes	
**ELIATM CALPROTECTIN TEST**	NAVIGENICS INC.	Calprotectin	Feces	IBD		
**EXAIBD™**	EXAGEN DIAGNOSTICS	Gene panel	White blood cells	IBD		
**EXAUC/CD™**	EXAGEN DIAGNOSTICS	Gene panel	White blood cells	UC vs. CD		
**FECAL MPO SAMPLE COLLECTION KIT**	EPITOPE DIAGNOSTICS	Myeloperoxidase (MPO)	Feces	IBD inflammation		

**GLYCOMINDS IBDX®**	GLYCOMINDS	ASCA, ALCA, ACCA, AMCA, Anti-L, Anti-C	Serum	IBD, UC vs. CD	Yes
**HUMAN CROHN'S DISEASE RT2 PROFILER PCR ARRAY**	MOLECULAR STAGING	84 gene panel		Research	Yes
**HUMAN FECAL NGAL (LCN2) ELISA KIT**	EPITOPE DIAGNOSTICS	NGAL, LCN2	Feces	IBD	Yes
**IBD BIOCHIP**	UNIVERSITY OF CALIFORNIA	C-reactive protein and calprotectin	Feces	IBD	
**IBD SGI DIAGNOSTIC®**	PROMETHEUS LAB INC.	Combined protein and genetic markers	Serum/ whole blood	IBD, UC vs. CD	Yes
**MONITR™**	PROMETHEUS LAB INC.	13 protein biomarkers	Serum	Mucosal healing in CD	Yes
**PROMETHEUS® ANSER® VDZ TEST**	PROMETHEUS LAB INC.	Antidrug antibodies	Serum	Treatment resistance	Yes
**Q-FOB™ QUANTITATIVE FECAL OCCULT BLOOD TEST KIT**	EPITOPE DIAGNOSTICS	Hemoglobin	Feces	IBD	Yes
**QUANTITATIVE FECAL CALPROTECTIN ELISA KIT**	EPITOPE DIAGNOSTICS	Calprotectin	Feces	IBD	Yes
**QUANTITATIVE FECAL/URINE MYELOPEROXIDASE ELISA KIT**	EPITOPE DIAGNOSTICS	MPO	Feces, Urine	Gut inflammation	
**RAID-CD**	GOODGUT	Specific biomarkers	Feces	CD	Yes

References

1. Starr, A.E.; Deeke, S.A.; Ning, Z.; Chiang, C.-K.; Zhang, X.; Mottawea, W.; Singleton, R.; Benchimol, E.I.; Wen, M.; Mack, D.R. Proteomic analysis of ascending colon biopsies from a paediatric inflammatory bowel disease inception cohort identifies protein biomarkers that differentiate crohn's disease from uc. *Gut* **2016**, gutjnl-2015-310705.
2. Li, N.; Wang, X.; Zhang, Y.; Zhai, J.; Zhang, T.; Wei, K. Comparative proteomics analysis of serum proteins in ulcerative colitis patients. *Molecular biology reports* **2012**, *39*, 5659–5667.
3. Vaiopoulou, A.; Gazouli, M.; Papadopoulou, A.; Anagnostopoulos, A.K.; Karamanolis, G.; Theodoropoulos, G.E.; M'Koma, A.; Tsangaris, G.T. Serum protein profiling of adults and children with crohn disease. *Journal of pediatric gastroenterology and nutrition* **2015**, *60*, 42–47.
4. Townsend, P.; Zhang, Q.; Shapiro, J.; Webb-Robertson, B.J.; Bramer, L.; Schepmoes, A.A.; Weitz, K.K.; Mallette, M.; Moniz, H.; Bright, R., et al. Serum proteome profiles in stricturing crohn's disease: A pilot study. *Inflammatory bowel diseases* **2015**, *21*, 1935–1941.
5. Wasinger, V.C.; Yau, Y.; Duo, X.; Zeng, M.; Campbell, B.; Shin, S.; Luber, R.; Redmond, D.; Leong, R.W. Low mass blood peptides discriminative of inflammatory bowel disease (ibd) severity: A quantitative proteomic perspective. *Molecular & cellular proteomics : MCP* **2016**, *15*, 256–265.
6. Yau, Y.Y.; Leong, R.W.L.; Pudipeddi, A.; Redmond, D.; Wasinger, V.C. Serological epithelial component proteins identify intestinal complications in crohn's disease. *Molecular & cellular proteomics : MCP* **2017**, *16*, 1244–1257.
7. Fan, F.; Mundra, P.A.; Fang, L.; Galvin, A.; Moore, X.L.; Weir, J.M.; Wong, G.; White, D.A.; Chin-Dusting, J.; Sparrow, M.P., et al. Lipidomic profiling in inflammatory bowel disease: Comparison between ulcerative colitis and crohn's disease. *Inflamm Bowel Dis* **2015**, *21*, 1511–1518.
8. Seeley, E.H.; Washington, M.K.; Caprioli, R.M.; M'Koma, A.E. Proteomic patterns of colonic mucosal tissues delineate crohn's colitis and ulcerative colitis. *PROTEOMICS-Clinical Applications* **2013**, *7*, 541–549.
9. M'koma, A.E.; Seeley, E.H.; Washington, M.K.; Schwartz, D.A.; Muldoon, R.L.; Herline, A.J.; Wise, P.E.; Caprioli, R.M. Proteomic profiling of mucosal and submucosal colonic tissues yields protein signatures that differentiate the inflammatory colitides. *Inflammatory bowel diseases* **2011**, *17*, 875–883.
10. Han, N.Y.; Choi, W.; Park, J.M.; Kim, E.H.; Lee, H.; Hahm, K.B. Label-free quantification for discovering novel biomarkers in the diagnosis and assessment of disease activity in inflammatory bowel disease. *Journal of digestive diseases* **2013**, *14*, 166–174.
11. Deeke, S.A.; Starr, A.E.; Ning, Z.; Ahmadi, S.; Zhang, X.; Mayne, J.; Chiang, C.K.; Singleton, R.; Benchimol, E.I.; Mack, D.R., et al. Mucosal-luminal interface proteomics reveals biomarkers of pediatric inflammatory bowel disease-associated colitis. *Am J Gastroenterol* **2018**, *113*, 713–724.
12. Zheng, X.; Chen, F.; Zhang, Q.; Liu, Y.; You, P.; Sun, S.; Lin, J.; Chen, N. Salivary exosomal psma7: A promising biomarker of inflammatory bowel disease. *Protein & cell* **2017**, *8*, 686–695.
13. Viennois, E.; Baker, M.T.; Xiao, B.; Wang, L.; Laroui, H.; Merlin, D. Longitudinal study of circulating protein biomarkers in inflammatory bowel disease. *Journal of proteomics* **2015**, *112*, 166–179.
14. Pouillon, L.; Bossuyt, P.; Peyrin-Biroulet, L. Considerations, challenges and future of anti-tnf therapy in treating inflammatory bowel disease. *Expert opinion on biological therapy* **2016**, *16*, 1277–1290.
15. Meuwis, M.-A.; Fillet, M.; Lutteri, L.; Marée, R.; Geurts, P.; De Seny, D.; Malaise, M.; Chapelle, J.-P.; Wehenkel, L.; Belaiche, J. Proteomics for prediction and characterization of response to infliximab in crohn's disease: A pilot study. *Clinical biochemistry* **2008**, *41*, 960–967.

16. Gazouli, M.; Anagnostopoulos, A.K.; Papadopoulou, A.; Vaiopoulou, A.; Papamichael, K.; Mantzaris, G.; Theodoropoulos, G.E.; Anagnou, N.P.; Tsangaris, G.T. Serum protein profile of crohn's disease treated with infliximab. *Journal of Crohn's & colitis* **2013**, *7*, e461-470.
17. Magnusson, M.K.; Strid, H.; Isaksson, S.; Bajor, A.; Lasson, A.; Ung, K.A.; Ohman, L. Response to infliximab therapy in ulcerative colitis is associated with decreased monocyte activation, reduced ccl2 expression and downregulation of tenascin c. *Journal of Crohn's & colitis* **2015**, *9*, 56–65.
18. Heier, C.R.; Fiorillo, A.A.; Chaisson, E.; Gordish-Dressman, H.; Hathout, Y.; Damsker, J.M.; Hoffman, E.P.; Conklin, L.S. Identification of pathway-specific serum biomarkers of response to glucocorticoid and infliximab treatment in children with inflammatory bowel disease. *Clinical and translational gastroenterology* **2016**, *7*, e192.
19. Poulsen, N.A.; Andersen, V.; Moller, J.C.; Moller, H.S.; Jessen, F.; Purup, S.; Larsen, L.B. Comparative analysis of inflamed and non-inflamed colon biopsies reveals strong proteomic inflammation profile in patients with ulcerative colitis. *BMC gastroenterology* **2012**, *12*, 76.
20. Barcelo-Batllori, S.; Andre, M.; Servis, C.; Levy, N.; Takikawa, O.; Michetti, P.; Reymond, M.; Felley-Bosco, E. Proteomic analysis of cytokine induced proteins in human intestinal epithelial cells: Implications for inflammatory bowel diseases. *Proteomics* **2002**, *2*, 551–560.
21. Sewell, G.W.; Hannun, Y.A.; Han, X.; Koster, G.; Bielawski, J.; Goss, V.; Smith, P.J.; Rahman, F.Z.; Vega, R.; Bloom, S.L., et al. Lipidomic profiling in crohn's disease: Abnormalities in phosphatidylinositols, with preservation of ceramide, phosphatidylcholine and phosphatidylserine composition. *The international journal of biochemistry & cell biology* **2012**, *44*, 1839–1846.
22. Li, X.; LeBlanc, J.; Elashoff, D.; McHardy, I.; Tong, M.; Roth, B.; Ippoliti, A.; Barron, G.; McGovern, D.; McDonald, K. Microgeographic proteomic networks of the human colonic mucosa and their association with inflammatory bowel disease. *CMGH Cellular and Molecular Gastroenterology and Hepatology* **2016**, *2*, 567–583.
23. Riaz, T.; Sollid, L.M.; Olsen, I.; de Souza, G.A. Quantitative proteomics of gut-derived th1 and th1/th17 clones reveal the presence of cd28+ nkg2d- th1 cytotoxic cd4+ t cells. *Molecular & cellular proteomics : MCP* **2016**, *15*, 1007–1016.
24. Shkoda, A.; Werner, T.; Daniel, H.; Gunckel, M.; Rogler, G.; Haller, D. Differential protein expression profile in the intestinal epithelium from patients with inflammatory bowel disease. *Journal of proteome research* **2007**, *6*, 1114–1125.
25. Nanni, P.; Mezzanotte, L.; Roda, G.; Caponi, A.; Levander, F.; James, P.; Roda, A. Differential proteomic analysis of ht29 cl.16e and intestinal epithelial cells by lc esi/qtof mass spectrometry. *Journal of proteomics* **2009**, *72*, 865–873.
26. Lochmatter, C.; Fischer, R.; Charles, P.D.; Yu, Z.; Powrie, F.; Kessler, B.M. Integrative phosphoproteomics links il-23r signaling with metabolic adaptation in lymphocytes. *Scientific reports* **2016**, *6*, 24491.
27. Hsieh, S.Y.; Shih, T.C.; Yeh, C.Y.; Lin, C.J.; Chou, Y.Y.; Lee, Y.S. Comparative proteomic studies on the pathogenesis of human ulcerative colitis. *Proteomics* **2006**, *6*, 5322–5331.
28. Bennike, T.B.; Carlsen, T.G.; Ellingsen, T.; Bonderup, O.K.; Glerup, H.; Bogsted, M.; Christiansen, G.; Birkelund, S.; Stensballe, A.; Andersen, V. Neutrophil extracellular traps in ulcerative colitis: A proteome analysis of intestinal biopsies. *Inflamm Bowel Dis* **2015**, *21*, 2052–2067.
29. Masoodi, M.; Pearl, D.S.; Eiden, M.; Shute, J.K.; Brown, J.F.; Calder, P.C.; Trebble, T.M. Altered colonic mucosal polyunsaturated fatty acid (pufa) derived lipid mediators in ulcerative colitis: New insight into relationship with disease activity and pathophysiology. *PLoS ONE* **2013**, *8*, e76532.

30. Schniers, A.; Anderssen, E.; Fenton, C.G.; Goll, R.; Pasing, Y.; Paulssen, R.H.; Florholmen, J.; Hansen, T. The proteome of ulcerative colitis in colon biopsies from adults-optimized sample preparation and comparison with healthy controls. *PROTEOMICS-Clinical Applications* **2017**.
31. Mottawea, W.; Chiang, C.-K.; Mühlbauer, M.; Starr, A.E.; Butcher, J.; Abujamel, T.; Deeke, S.A.; Brandel, A.; Zhou, H.; Shokralla, S. Altered intestinal microbiota–host mitochondria crosstalk in new onset crohn's disease. *Nature Communications* **2016**, *7*.
32. Ehehalt, R.; Wagenblast, J.; Erben, G.; Lehmann, W.D.; Hinz, U.; Merle, U.; Stremmel, W. Phosphatidylcholine and lysophosphatidylcholine in intestinal mucus of ulcerative colitis patients. A quantitative approach by nanoelectrospray-tandem mass spectrometry. *Scand J Gastroenterol* **2004**, *39*, 737–742.
33. Erickson, A.R.; Cantarel, B.L.; Lamendella, R.; Darzi, Y.; Mongodin, E.F.; Pan, C.; Shah, M.; Halfvarson, J.; Tysk, C.; Henrissat, B. Integrated metagenomics/metaproteomics reveals human host-microbiota signatures of crohn's disease. *PLoS ONE* **2012**, *7*, e49138.
34. Duguet, F.; Locard-Paulet, M.; Marcellin, M.; Chaoui, K.; Bernard, I.; Andreoletti, O.; Lesourne, R.; Burlet-Schiltz, O.; Gonzalez de Peredo, A.; Saoudi, A. Proteomic analysis of regulatory t cells reveals the importance of themis1 in the control of their suppressive function. *Molecular & cellular proteomics : MCP* **2017**.
35. Bertrand, J.; Tennoune, N.; Marion-Letellier, R.; Goichon, A.; Chan, P.; Mbodji, K.; Vaudry, D.; Dechelotte, P.; Coeffier, M. Evaluation of ubiquitinated proteins by proteomics reveals the role of the ubiquitin proteasome system in the regulation of grp75 and grp78 chaperone proteins during intestinal inflammation. *Proteomics* **2013**, *13*, 3284–3292.
36. Schaubeck, M.; Clavel, T.; Calasan, J.; Lagkouvardos, I.; Haange, S.B.; Jehmlich, N.; Basic, M.; Dupont, A.; Hornef, M.; von Bergen, M., et al. Dysbiotic gut microbiota causes transmissible crohn's disease-like ileitis independent of failure in antimicrobial defence. *Gut* **2016**, *65*, 225–237.
37. Mayers, M.D.; Moon, C.; Stupp, G.S.; Su, A.I.; Wolan, D.W. Quantitative metaproteomics and activity-based probe enrichment reveals significant alterations in protein expression from a mouse model of inflammatory bowel disease. *Journal of proteome research* **2017**, *16*, 1014–1026.
38. Lee, Y.; Choo, J.; Kim, S.J.; Heo, G.; Pothoulakis, C.; Kim, Y.H.; Im, E. Analysis of endogenous lipids during intestinal wound healing. *PLoS ONE* **2017**, *12*.
39. Samie, M.; Lim, J.; Verschueren, E.; Baughman, J.M.; Peng, I.; Wong, A.; Kwon, Y.; Senbabaoglu, Y.; Hackney, J.A.; Keir, M., et al. Selective autophagy of the adaptor trif regulates innate inflammatory signaling. *Nature immunology* **2018**, *19*, 246–254.
40. Di Narzo, A.F.; Telesco, S.E.; Brodmerkel, C.; Argmann, C.; Peters, L.A.; Li, K.; Kidd, B.; Dudley, J.; Cho, J.; Schadt, E.E., et al. High-throughput characterization of blood serum proteomics of ibd patients with respect to aging and genetic factors. *PLoS genetics* **2017**, *13*, e1006565.
41. Suhre, K.; Arnold, M.; Bhagwat, A.M.; Cotton, R.J.; Engelke, R.; Raffler, J.; Sarwath, H.; Thareja, G.; Wahl, A.; DeLisle, R.K. Connecting genetic risk to disease end points through the human blood plasma proteome. *Nature communications* **2017**, *8*, 14357.
42. Barnett, M.P.; Cooney, J.M.; Dommels, Y.E.; Nones, K.; Brewster, D.T.; Park, Z.; Butts, C.A.; McNabb, W.C.; Laing, W.A.; Roy, N.C. Modulation of colonic inflammation in mdr1a(-/-) mice by green tea polyphenols and their effects on the colon transcriptome and proteome. *The Journal of nutritional biochemistry* **2013**, *24*, 1678–1690.
43. Cooney, J.M.; Barnett, M.P.; Dommels, Y.E.; Brewster, D.; Butts, C.A.; McNabb, W.C.; Laing, W.A.; Roy, N.C. A combined omics approach to evaluate the effects of dietary curcumin on colon inflammation in the mdr1a(-/-) mouse model of inflammatory bowel disease. *The Journal of nutritional biochemistry* **2016**, *27*, 181–192.

44. Wang, R.; Gu, X.; Dai, W.; Ye, J.; Lu, F.; Chai, Y.; Fan, G.; Gonzalez, F.J.; Duan, G.; Qi, Y. A lipidomics investigation into the intervention of celastrol in experimental colitis. *Molecular bioSystems* **2016**, *12*, 1436–1444.
